# Evaluation of nucleic acid-based matrix-assisted laser desorption/ionization time-of-flight mass spectrometry for rapid identification of *Mycobacterium tuberculosis* and drug resistance in patients with retreatment tuberculosis

**DOI:** 10.3389/fmicb.2026.1694247

**Published:** 2026-06-18

**Authors:** Jiajun Zhou, Xiaoqing Lin, Yunan Zhang, Liufeng Qiu, Saiduo Liu, Zhengxing Wu, Yueying Zhou, Ning Pan, Chaochao Qiu, Jichan Shi

**Affiliations:** 1 Department of Infection, Wenzhou Central Hospital, Dingli Clinical College of Wenzhou Medical University, Wenzhou, Zhejiang, China; 2 Shanghai University, Shanghai, China; 3 Department of Infection, Wenzhou Central Hospital, Postgraduate Training Base Alliance of Wenzhou Medical University, Wenzhou, Zhejiang, China

**Keywords:** drug resistance, matrix-assisted laser desorption ionization time-of-flight mass spectrometry, *Mycobacterium tuberculosis*, nucleic acid detection, pulmonary, retreatment failure, tuberculosis

## Abstract

**Objective:**

This study aimed to evaluate the value of nucleic acid-based matrix-assisted laser desorption/ionization time-of-flight mass spectrometry (MALDI-TOF MS) in identifying mycobacterial species and detecting drug resistance in patients with retreatment pulmonary tuberculosis (TB).

**Methods:**

The clinical data of 100 patients with retreatment pulmonary TB treated at Wenzhou Central Hospital between 1 June 2022 and 31 December 2023 were retrospectively collected and analyzed. Respiratory secretions were examined using BACTEC MGIT 960 Rapid Liquid Culture (MGIT 960 culture), drug-sensitive gene chip strain identification and nucleic acid-based MALDI-TOF MS assay (MassARRAY® system). Unlike traditional protein-based MALDI-TOF MS, this nucleic acid-based approach detects specific gene mutations associated with drug resistance. The results of the MGIT 960 culture and drug resistance testing served as the benchmark for evaluating the sensitivity, specificity, positive predictive value (PPV) and negative predictive value (NPV) of the MALDI-TOF MS assay.

**Results:**

For *Mycobacterium tuberculosis* (MTB) detection, compared with the MGIT 960 culture, the nucleic acid-based MALDI-TOF MS assay demonstrated a sensitivity of 97.01% (65/67), specificity of 63.64% (21/33), PPV of 84.42% (65/77) and NPV of 91.30% (21/23), with a concordance of 86.00% and a kappa value of 0.619. The difference in detection results between the two methods was statistically significant (χ^2^ = 15.625, *p* < 0.001). For drug-resistance detection in the 34 MTB-positive cases with both methods, concordance with MGIT 960 was 97% for rifampicin (97% sensitivity, 100% specificity) and 88% for isoniazid (86% sensitivity, 100% specificity), with overall *κ* = 0.83 across first- and second-line drugs.

**Conclusion:**

The nucleic acid-based MALDI-TOF MS assay demonstrates promising performance in rapidly identifying MTB and detecting drug resistance, serving as a valuable complementary tool for rapid clinical diagnosis and personalized medication guidance in patients with retreatment TB. The detection of non-tuberculous mycobacteria requires further validation with larger sample sizes.

## 1 Introduction

The current situation regarding the prevention and control of drug-resistant tuberculosis (TB) is particularly concerning. Several studies have indicated that the rate of drug resistance among retreatment TB cases is substantially higher than that observed in primary treatment cases (Antimicrobial Resistance Collaborators, 2022; Chinese Association for the Prevention of Tuberculosis, 2019). This suggests that patients undergoing retreatment are more prone to developing resistance to anti-TB drugs. The emergence of this resistance is associated not only with the duration of drug exposure (Alemu et al., 2023) but also with a variety of factors. These include irregularities in medication during treatment, adverse drug reactions, an increased number of previous treatment courses and poor patient adherence. The emergence of drug-resistant TB strains has substantially increased the complexity of treatment regimens, resulting in prolonged hospitalization and higher mortality rates (Antimicrobial Resistance Collaborators, 2022).

In recent years, matrix-assisted laser desorption ionization time-of-flight mass spectrometry (MALDI-TOF MS) has gained widespread recognition in microbial identification, particularly for *Mycobacterium tuberculosis* (MTB) (Wu et al., 2022). However, it is crucial to distinguish between two fundamentally different MALDI-TOF MS approaches. Traditional protein-based MALDI-TOF MS (such as MALDI Biotyper) identifies bacteria by analyzing ribosomal proteins, whereas nucleic acid-based MALDI-TOF MS detects specific genetic mutations associated with drug resistance. Recent studies during 2024–2025 have demonstrated the clinical utility of nucleic acid-based MALDI-TOF MS in TB diagnosis. Li et al. (2022) reported a sensitivity of 71.5% and a specificity of 85.5% for nucleotide MALDI-TOF MS in analyzing bronchoalveolar lavage fluid (BALF) from patients with suspected TB. Shi et al. (2025) evaluated nucleotide MALDI-TOF MS for mycobacterial species identification, showing high accuracy in distinguishing both MTB complex and non-tuberculous mycobacteria (NTM). Wu et al. (2022) demonstrated sensitivities of 92.2 and 90.9% for the detection of rifampicin (RIF) and isoniazid (INH) resistance, respectively. Ou et al. (2024) developed a comprehensive optimized nucleotide MALDI-TOF MS database for highly pathogenic bacteria, including mycobacteria and anti-tuberculosis drug resistance detection. Although these studies have shown promising results in primary TB cases, research specifically focusing on patients with retreatment TB, who exhibit higher rates of drug resistance, remains limited.

The principle behind MALDI-TOF MS can be applied to both protein and nucleic acid analysis. For mycobacterial identification, this study employs MassARRAY® nucleic acid MALDI technology (Agena Bioscience, San Diego, CA, USA), which differs from traditional protein-based MALDI-TOF MS (such as the MALDI Biotyper). The MassARRAY® system uses a primer extension coupled with time-of-flight mass spectrometry to detect specific nucleotide polymorphisms, enabling rapid identification of drug resistance-associated mutations in genes such as rpoB for RIF resistance and katG for INH resistance (Su et al., 2017). This nucleic acid-based approach offers distinct advantages for the detection of drug resistance in mycobacteria, as it enables simultaneous detection of multiple resistance-conferring mutations through multiplexed primer extension reactions (Su et al., 2017; Liu et al., 2025).

This study aims to evaluate the clinical utility of nucleic acid MALDI-TOF MS (MassARRAY®) in identifying MTB and detecting drug resistance, specifically in patients with retreatment pulmonary TB – a population with distinct characteristics and higher rates of drug resistance than treatment-naïve cases. Using MGIT 960 culture as a control, we assess the performance of MALDI-TOF MS in clinical settings to determine its potential as a diagnostic tool for personalized treatment strategies.

## 2 Methods

### 2.1 Study design and participants

This study included 100 patients with retreatment pulmonary TB who attended Wenzhou Central Hospital between 1 June 2022 and 31 December 2023.

#### 2.1.1 Sample size calculation

The sample size was calculated based on the expected sensitivity and specificity of MALDI-TOF MS in detecting drug-resistant *MTB*. According to a pilot study and previous literature, the sensitivity of MALDI-TOF MS for drug-resistant TB was estimated at 90% and the specificity at 85% (Liang et al., 2023). To achieve a 95% confidence level and a 5% margin of error, the following formula was used for sample size calculation: *n* = Z^2^P(1-P)/E^2^ where *n* is the required sample size, *Z* is the Z-value corresponding to the 95% confidence level (1.96), *P* is the estimated proportion of drug-resistant TB (0.90 for sensitivity and 0.85 for specificity) and *E* is the margin of error (5% or 0.05). For the primary outcome (sensitivity and specificity), the estimated sample size was 100 patients. An additional 10% was included to account for potential dropouts or missing data, resulting in a final target sample size of 110 patients.

The inclusion criteria were as follows: (1) patients who had failed initial treatment; (2) patients with sputum repositivity after completing a full course of regular medication; (3) patients who had received irregular chemotherapy for >1 month; and (4) patients with chronic bacterial excretion. Patients meeting any of these criteria were diagnosed with retreatment TB.

The exclusion criteria were as follows: (1) active malignancy; (2) severe cardiovascular, respiratory or hepatic disease that could interfere with treatment or outcomes; (3) known immunodeficiency, including HIV infection or current use of immunosuppressive therapy; (4) a previous history of multidrug-resistant (MDR) TB or extensively drug-resistant (XDR) TB not covered by the study’s drug resistance profile; (5) pregnancy or breastfeeding; and (6) concurrent participation in another clinical trial.

Retreatment TB was defined as follows: (1) failure of initial treatment; (2) sputum repositivity following completion of a full course of regular medication; (3) irregular chemotherapy extending beyond 1 month; and (4) chronic bacterial excretion.

The study protocol was approved by the Ethics Review Board of Wenzhou Central Hospital (approval number: 2021-03-074), and written informed consent was obtained from all participants.

### 2.2 Measures and definitions

#### 2.2.1 Reagents and instruments

The MassARRAY® nucleic acid time-of-flight mass spectrometry-based platform for rapid detection of *Mycobacterium* identification and drug resistance was developed by Shanghai Conlight Medical Co., Ltd. Roche culture medium and mycobacterial drug-sensitive Roche culture tubes were purchased from Zhuhai Baso Co., Ltd. The Langji L96+/Y-type polymerase chain reaction (PCR) instrument/gene amplification instrument was purchased from Hangzhou LongGene Scientific Instruments Co., Ltd. Roche diagnostic real-time PCR amplification instruments (models LC-480I and Z480) and the FYY-3 molecular hybridisation instrument were purchased from Yaneng BIOscience (Shenzhen) Co., Ltd. Adjustable pipettes (2–10 μL, 20–200 μL) were purchased from Ebende, Germany. The Fosun biosafety cabinet was purchased from Shanghai Lishen Scientific Instruments Co., Ltd. The chromogenic solution was freshly prepared using distilled water according to the required concentration.

#### 2.2.2 Ziehl–Neelsen acid-fast staining

A 5 mL sample of BALF was placed in a centrifuge tube and centrifuged at 3,000 rpm for 20 min. The supernatant was discarded, and the precipitate was collected and smeared onto a slide. The smears were air-dried and fixed by gentle heating with an alcohol lamp, followed by acid-fast staining.

#### 2.2.3 *Mycobacterium* culture and drug sensitivity test

*Mycobacterium* culture was performed using MGIT 960 culture. The BALF was thoroughly mixed, and 5 mL was placed in a centrifuge tube. A digestive solution was added. Specifically, 2 mL of N-acetyl-L-cysteine (NALC)–NaOH (final NaOH 1.0%, NALC 0.5%) was added to 5 mL BALF. The mixture was vortexed and oscillated until fully digested. The mixture was incubated for 15 min at room temperature, centrifuged at 3,000 × g for 15 min, and the pellet was resuspended in 1 mL phosphate buffer solution (PBS). The supernatant was discarded, and a suspension was prepared by adding a small amount of PBS to the precipitate and mixing thoroughly. The suspension was inoculated into MGIT culture tubes and incubated in the culture system. For MGIT tubes with positive culture results, acid-fast staining was repeated to confirm the presence of *Mycobacterium* and to proceed with strain identification. The H37Rv standard strain was used as a quality control, and the minimum inhibitory concentration drug sensitivity test was conducted for first-line drugs (RIF, INH, ethambutol [EMB], streptomycin [Sm]) and second-line drugs (fluoroquinolones, amikacin, kanamycin, capreomycin).

#### 2.2.4 Strain identification

Strain identification was performed using the Boao gene chip method, which enables accurate identification of *Mycobacterium* species based on nucleic acid hybridisation.

#### 2.2.5 MassARRAY® test

The MassARRAY® system used in this study was a nucleic acid-based MALDI-TOF MS platform that employs primer extension technology coupled with time-of-flight mass spectrometry. Unlike traditional protein-based MALDI-TOF MS (such as the MALDI-Biotyper), this system specifically detects nucleotide polymorphisms and mutations associated with drug resistance. For species identification, MassARRAY® targets rpoB, hsp65 and 16S rRNA amplicons; species-specific single-nucleotide polymorphisms are resolved by mass differences of the extension products (Zhang et al., 2025).

Specimen DNA extraction: DNA was isolated using the QIAamp DNA Mini Kit (Qiagen, Cat. No. 51304) according to the manufacturer’s protocol. The DNA was extracted from the specimen following the procedures outlined in the kit manual.

Nucleic acid detection: the sample DNA was mixed with a 5 μL PCR master mix consisting of 10 mM Tris–HCl, 50 mM KCl, 3 mM MgCl₂, 0.2 mM each deoxyribonucleoside triphosphate (dNTP) and 0.5 U Taq polymerase. The PCR amplification was conducted using an AB Veriti 96/384 amplifier under the following conditions: 95 °C for 2 min, 95 °C for 30 s, 60 °C for 30 s, 72 °C for 60 s (45 cycles) and 72 °C for 5 min. The reaction mixture was then treated with a shrimp alkaline phosphatase (SAP) solution to remove unconsumed dNTPs (37 °C for 40 min, followed by 85 °C for 5 min to terminate the reaction), and an extension reaction was subsequently performed.

A sample was considered MTB-positive in the MALDI-TOF MS assay if specific peaks corresponding to MTB-associated mutations were detected with a signal-to-noise ratio >3 and matched the reference database with a score ≥2.0.

#### 2.2.6 Polymerase chain reaction

The total reaction volume was 10 μL. The PCR reaction consisted of 3.3 μL deionized water, 1.0 μL 10 × PCR buffer with 20 mM MgCl₂, 0.8 μL 20 mM MgCl₂, 0.2 μL 25 mM deoxynucleotide triphosphate mix, 1.0 μL 1 μM primer mix, 0.4 μL 5 U/μL PCR enzyme and 3.3 μL 5 ng/μL DNA template. The PCR reaction was performed under the following conditions: 95 °C for 2 min, followed by 30 cycles of 95 °C for 30 s, 56 °C for 30 s and 72 °C for 60 s, with a final extension at 72 °C for 5 min. A blank control (2 μL deionized water), a negative control (2 μL TE buffer) and a positive control were included in each run.

#### 2.2.7 Shrimp alkaline phosphatase reaction

The SAP mix was prepared to a final volume of 2 μL, consisting of 1.53 μL deionized water, 0.17 μL SAP buffer and 0.30 μL SAP enzyme. The mixture was added to the PCR product and incubated at 37 °C for 40 min, followed by inactivation at 85 °C for 5 min.

#### 2.2.8 Extension reaction

A 2.0 μL iPLEX extension reaction mixture containing 0.62 μL deionized water, 0.20 μL iPLEX buffer, 0.20 μL iPLEX termination mix, 0.94 μL Extend primer mix and 0.04 μL iPLEX enzyme was added to each well. The extension reaction was performed as follows: denaturation at 94 °C for 30 s, followed by 40 cycles of 94 °C for 5 s, 52 °C for 5 s, 80 °C for 5 s (MassARRAY® iPLEX default cycling profile) and a final extension at 72 °C for 3 min.

#### 2.2.9 Mass spectrometry analysis

**Figure 1:**
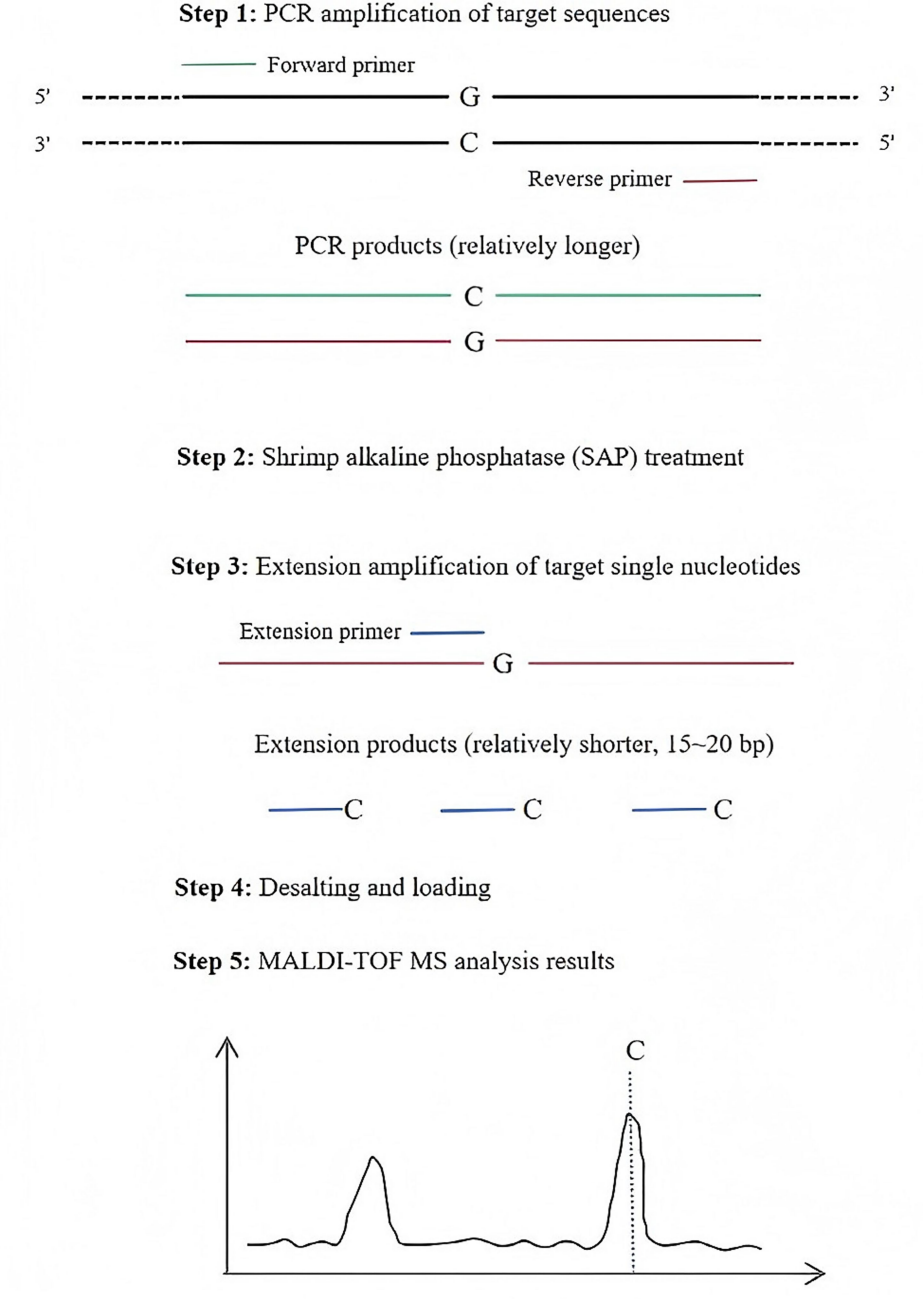
The flowchart of nucleotide MALDI-TOF-MS. MALDI-TOF-MS, nucleotide matrix-assisted laser desorption/ionization time-of-flight mass spectrometry. MALDI-TOF MS, matrix-assisted laser desorption/ionization time-of-flight mass spectrometry; DNA, deoxyribonucleic acid; PCR, polymerase chain reaction; SAP, shrimp alkaline phosphatase; MS, mass spectrometry.

Polymerase chain reaction products were spotted on the chip using the MassARRAY® Nanodispenser (Agena Bioscience, San Diego, CA, USA). The chip was then placed into the MassARRAY® Analyzer for detection and analysis (Figure 1). Spectra were acquired in linear positive mode (m/z 2,000–12,000) with a 60 Hz laser, 500 shots per spectrum.

#### 2.2.10 Quality control measures for consistency and reliability

Sample processing: biological samples (including BALF) were processed according to standardized protocols, transported to the laboratory immediately, stored at −80 °C and thawed under sterile conditions prior to testing.

Controls: each test batch included positive controls (H37Rv strain) and negative controls (sterile saline) to confirm the accuracy and prevent contamination.

H37Rv standard strain: the H37Rv strain was used for quality control in MTB identification and drug resistance testing. It was cultured alongside patient samples and compared with the reference database.

Calibration and maintenance: the MALDI-TOF MS equipment was calibrated before each batch and maintained regularly to ensure accuracy and reduce technical errors.

Data analysis: mass spectra were analyzed using standard software and cross-verified by independent analysts. Any discrepancies were resolved through retesting.

Bias control: blind testing was employed, with technicians blinded to sample treatment groups. Samples were randomly coded to minimize observer bias and ensure objectivity.

### 2.3 Data analysis

#### 2.3.1 Statistical analysis and diagnostic performance evaluation

*Statistical analysis*: Data were analyzed using SPSS 22.0. Continuous data were tested for normality using the Shapiro–Wilk test. Normally distributed data were compared using t-tests, whereas non-normally distributed data were analyzed using the Mann–Whitney U test. Categorical data were compared using χ^2^ tests for independent comparisons, whereas McNemar’s test was used for paired comparisons between the MALDI-TOF MS and MGIT 960 culture results. A *p*-value of <0.05 was considered statistically significant. For the comparison of baseline characteristics, patients were divided into two groups based on the MALDI-TOF MS results: positive (*n* = 77) and negative (*n* = 23). The reported *p*-values indicate the strength of association between the two methods rather than a difference between them.

*Diagnostic performance*: the MALDI-TOF MS assay was evaluated for sensitivity, specificity, positive predictive value (PPV), negative predictive value (NPV), concordance rate and kappa value. The MGIT 960 culture and gene chip results served as references. Confidence intervals were calculated for each parameter to assess precision.

## 3 Results

### 3.1 Characteristics of enrolled patients

**Figure 2:**
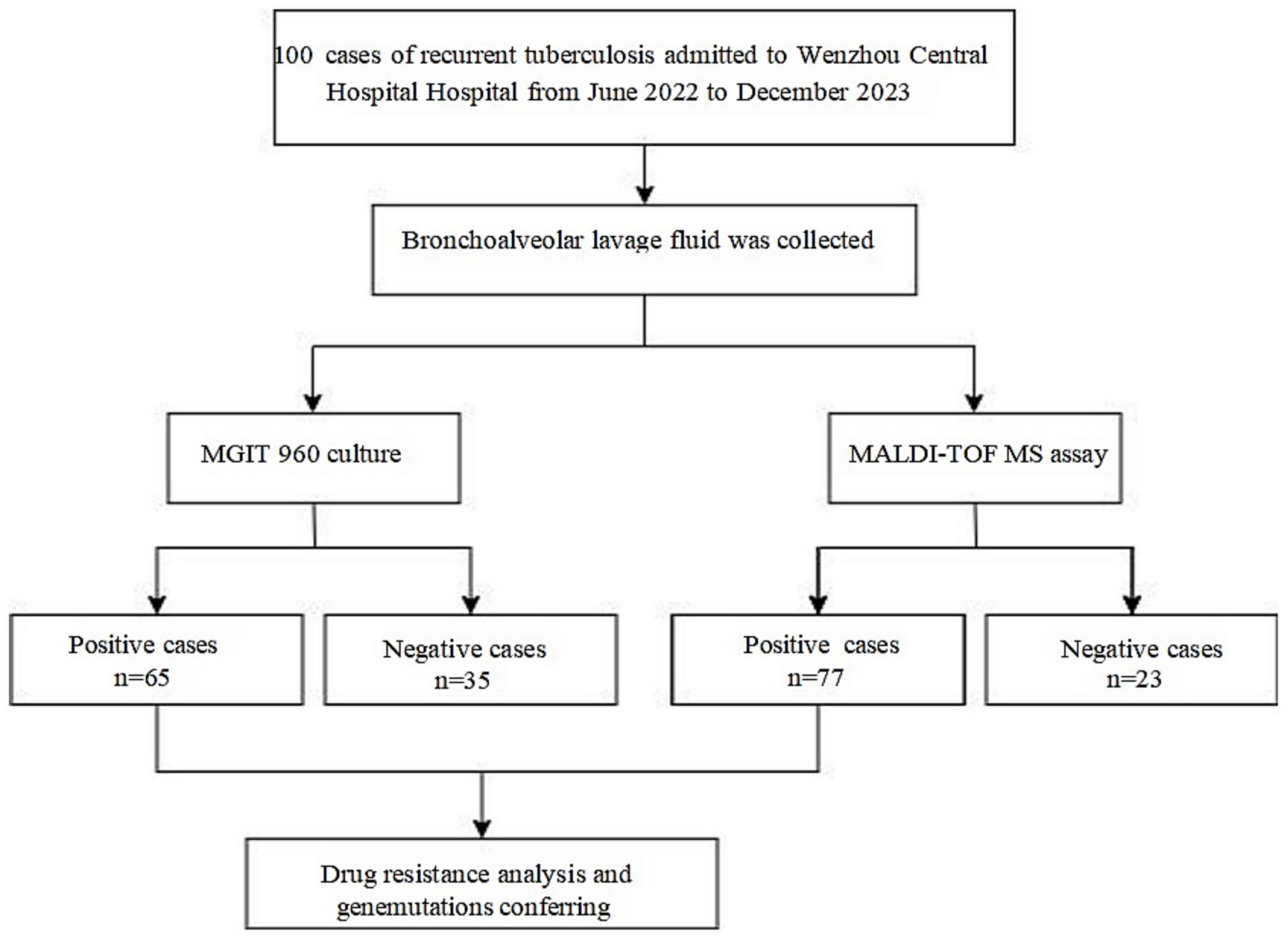
Flow diagram of the study design. BALF, bronchoalveolar lavage fluid; MTB, *Mycobacterium tuberculosis*; NTM, nontuberculous mycobacteria; MGIT 960, Mycobacteria Growth Indicator Tube 960 liquid culture; MALDI-TOF MS, matrix-assisted laser desorption/ionization time-of-flight mass spectrometry.

The flow chart of the study design is shown in Figure 2. Among the 100 patients with retreatment TB, 60 (60%) were male and 40 (40%) were female. The mean age was 50.5 ± 15.7 years, and the mean body mass index was 21.4 ± 4.0. A total of 80 patients had a history of underlying medical conditions, including 19 cases of diabetes mellitus, 17 cases of hypertension, 9 cases of chronic hepatitis B, 2 cases of silicosis, 5 cases of hyperlipidaemia, 3 cases of chronic obstructive pulmonary disease, 2 cases of asthma and 3 cases of long-term hormone use (gouty arthritis, undifferentiated connective tissue disease, systemic erythrocyte disorder).

With regard to chest computed tomography imaging, 24 patients had cavitation, 15 had pleural effusion and 17 had bronchiectasis. The baseline characteristics of the patients are summarized in Table 1. The MALDI-TOF MS positive group (*n* = 77) and negative group (*n* = 23) were comparable in terms of age, sex, previous treatment history, smoking status and clinical symptoms (*p* > 0.05 for all). There were no significant differences between the two groups with respect to the duration of TB or the presence of comorbidities (Table 1). All patients in both groups had a history of at least one previous TB treatment, and the majority had a relapse of TB.

**Table 1:** Descriptive statistics of retreatment tuberculosis patients (*N* = 100).

Characteristics	x ± SD	*n* (%)
Age	50.5 ± 15.7	
BMI	21.4 ± 4.0	
Gender		
Male		60 (60%)
Female		40 (40%)
Underlying diseases		
Diabetes		19 (19%)
Hypertension		17 (17%)
Chronic hepatitis B		9 (9%)
Silicosis		2 (2%)
Hyperlipidaemia		5 (5%)
Chronic obstructive pulmonary disease		3 (3%)
Asthma		2 (2%)
Glucocorticoids treatment		3 (3%)
Concomitant symptom		
Cough		20 (20%)
Fever		5 (5%)
Hemoptysis		7 (7%)
Chest pain		3 (3%)
Chest computed tomography scan		
Cavity		24 (24%)
Pleural effusion		15 (15%)
Bronchiectasis		17 (17%)

### 3.2 Efficacy of nucleic acid-based matrix-assisted laser desorption ionization time-of-flight mass spectrometry assay in detecting *Mycobacterium tuberculosis*

Of the 100 patients, 67 cases tested positive using the MGIT 960 liquid culture, whereas 77 tested positive using the nucleic acid-based MALDI-TOF MS assay. Among the 77 MALDI-TOF MS positive cases, 71 were identified as MTB and 6 were identified as NTM. However, the limited number of NTM cases (*n* = 6) precludes definitive conclusions regarding NTM detection. To accurately assess the performance of MALDI-TOF MS specifically for MTB detection, we focused our analysis on MTB cases.

The MGIT 960 culture was used as the gold standard to evaluate the detection efficacy of the nucleic acid-based MALDI-TOF MS assay for MTB. Among the 67 MGIT 960 culture-positive cases, 65 were also positive by MALDI-TOF MS (including only MTB, excluding NTM cases for this calculation), and 2 were negative. Among the 33 MGIT 960 culture-negative cases, 12 were positive by MALDI-TOF MS and 21 were negative. The results indicated that the sensitivity of the MALDI-TOF MS assay for detecting MTB was 97.01% (65/67), and the specificity was 63.64% (21/33). The PPV and NPV were 84.42% (65/77) and 91.30% (21/23), respectively. The MALDI-TOF MS results were strongly associated with the MGIT 960 results (McNemar’s χ^2^ = 15.625, *p* < 0.001), indicating that the assay performed substantially better than chance and closely aligned with the gold standard (Table 2).

**Table 2:** Detection efficacy of *Mycobacterium tuberculosis* identification by nucleic acid-based MALDI-TOF MS compared with MGIT 960 culture (*N* = 100).

A. Contingency			
MALDI-TOF MS result	MGIT 960 culture		Total
	Positive	Negative	
Positive	65	12	77
Negative	2	21	23
Total	67	33	100

B. Performance metrics		
Metric	Value	95% confidence interval
Sensitivity	97.01%	89.63–99.64%
Specificity	63.64%	45.12–79.60%
PPV	84.42%	74.05–91.71%
NPV	91.30%	71.96–98.93%
Accuracy	86.00%	77.63–92.13%
Kappa (*κ*)	0.619	0.454–0.784

Statistical test: McNemar’s χ^2^ = 15.625, *p* < 0.001.

PPV, positive predictive value; NPV, negative predictive value. MGIT 960 culture was used as the gold standard. Confidence intervals were calculated using the Wilson score method. Analysis focused on MTB detection specifically (*n* = 100 patients; 67 MGIT 960 positive, 77 MALDI-TOF MS positive including 71 MTB and 6 NTM).

Among the 12 MALDI-positive/culture-negative cases, 6 were identified as NTM by MALDI-TOF MS. The gene chip confirmed 4 of these NTM, which represents a distinct clinical entity from MTB. For the remaining 6 MTB-positive/culture-negative cases, 2 were detected only by MALDI-TOF MS. This discordance may be attributed to several factors: the higher analytic sensitivity of nucleic acid-based detection methods for identifying non-viable organisms or fragmented mycobacterial DNA, low bacterial loads in BALF samples, or the presence of paucibacillary disease where culture sensitivity is limited.

### 3.3 Nucleic acid-based matrix-assisted laser desorption ionization time-of-flight mass spectrometry assay for drug resistance identification

For drug resistance analysis, we focused on the 34 cases that were MTB-positive by both MGIT 960 culture and MALDI-TOF MS assay, as these cases allow for direct comparison of drug susceptibility testing results between the two methods. This subset represents patients with retreatment TB confirmed MTB infection by both methodologies, ensuring the validity of drug resistance comparisons. Among these 34 cases, MALDI-TOF MS detected drug resistance in 45 total instances across different drugs, all identified as MTB.

The consistency analysis of different drug resistances is shown in Table 3. For RIF resistance, the concordance between MGIT 960 culture and MALDI-TOF MS was 97.06% (33/34), with sensitivity 96.97% (32/33), specificity 100% (1/1), PPV 100% (32/32) and NPV 50% (1/2). The difference was statistically significant (χ^2^ = 16.485, *p* < 0.001), indicating strong agreement between the methods, with a kappa value of 0.65.

**Table 3:** Consistency analysis of drug resistance detection between nucleic acid-based MALDI-TOF MS and MGIT 960 culture (*n* = 34 MTB-positive cases).

A. First-line drugs			
1. Rifampicin (RIF)			
MALDI-TOF MS	MGIT 960 culture		Total
	Resistant	Sensitive	
Resistant	32	0	32
Sensitive	1	1	2
Total	33	1	34
Performance metrics: Sensitivity: 96.97% (95% CI: 84.24–99.92%); Specificity: 100% (95% CI: 2.50–100%); PPV: 100% (95% CI: 89.11–100%); NPV: 50.00% (95% CI: 1.26–98.74%); Accuracy: 97.06%, κ = 0.65, χ^2^ = 16.485, *p* < 0.001.			

2. Isoniazid (INH)			
MALDI-TOF MS	MGIT 960 culture		Total
	Resistant	Sensitive	
Resistant	25	0	25
Sensitive	4	5	9
Total	29	5	34
Performance metrics: Sensitivity: 86.21% (95% CI: 68.34–96.11%). Specificity: 100% (95% CI: 47.82–100%). PPV: 100% (95% CI: 86.28–100%). NPV: 55.56% (95% CI: 21.20–86.30%). Accuracy: 88.24%, κ = 0.64, χ^2^ = 16.284, *p* < 0.001.			

3. Ethambutol (EMB)			
MALDI-TOF MS	MGIT 960 culture		Total
	Resistant	Sensitive	
Resistant	9	3	12
Sensitive	1	21	22
Total	10	24	34
Performance metrics: Sensitivity: 90.00% (95% CI: 55.50–99.75%); Specificity: 87.50% (95% CI: 67.64–97.34%); PPV: 75.00% (95% CI: 42.81–94.51%); NPV: 95.45% (95% CI: 77.16–99.88%); Accuracy: 88.24%, κ = 0.73, χ^2^ = 18.565, *p* < 0.001.			

4. Streptomycin (Sm)			
MALDI-TOF MS	MGIT 960 culture		Total
	Resistant	Sensitive	
Resistant	18	0	18
Sensitive	1	15	16
Total	19	15	34
Performance metrics: Sensitivity: 94.74% (95% CI: 73.97–99.87%); Specificity: 100% (95% CI: 78.20–100%); PPV: 100% (95% CI: 81.47–100%); NPV: 93.75% (95% CI: 69.77–99.84%); Accuracy: 97.06%, κ = 0.94, χ^2^ = 30.197, *p* < 0.001.			

B. Second-line drugs			
1. Levofloxacin (Ofx)			
MALDI-TOF MS	MGIT 960 culture		Total
	Resistant	Sensitive	
Resistant	8	0	8
Sensitive	0	26	26
Total	8	26	34
Performance metrics: Sensitivity: 100% (95% CI: 63.06–100%); Specificity: 100% (95% CI: 86.78–100%); PPV: 100% (95% CI: 63.06–100%); NPV: 100% (95% CI: 86.78–100%); Accuracy: 100%, κ = 1.00, χ^2^ = 34.000, *p* < 0.001.			

2. Moxifloxacin (Mfx)			
MALDI-TOF MS	MGIT 960 culture		Total
	Resistant	Sensitive	
Resistant	8	0	8
Sensitive	0	26	26
Total	8	26	34
Performance metrics: Sensitivity: 100% (95% CI: 63.06–100%); Specificity: 100% (95% CI: 86.78–100%); PPV: 100% (95% CI: 63.06–100%); NPV: 100% (95% CI: 86.78–100%); Accuracy: 100%, κ = 1.00, χ^2^ = 34.000, *p* < 0.001.			

3. Amikacin/kanamycin/Capreomycin (am/km/cm)			
MALDI-TOF MS	MGIT 960 culture		Total
	Resistant	Sensitive	
Resistant	2	1	3
Sensitive	0	31	31
Total	2	32	34
Performance metrics: Sensitivity: 100% (95% CI: 15.81–100%); Specificity: 96.88% (95% CI: 83.78–99.92%); PPV: 66.67% (95% CI: 9.43–99.16%); NPV: 100% (95% CI: 88.78–100%); Accuracy: 97.06%, κ = 0.78, χ^2^ = 21.958, *p* < 0.001.			

PPV, positive predictive value; NPV, negative predictive value; κ, kappa coefficient; CI, confidence interval; RIF, rifampicin; INH, isoniazid; EMB, ethambutol; Sm, streptomycin; Ofx, levofloxacin; Mfx, moxifloxacin; am, amikacin; km, kanamycin; cm, capreomycin.

For INH resistance, the concordance was 88.24% (30/34), sensitivity 86.21% (25/29), specificity 100% (5/5), PPV 100% (25/25) and NPV 55.56% (5/9). The difference was statistically significant (χ^2^ = 16.284, *p* < 0.001), with a kappa value of 0.64. For EMB resistance, the concordance was 88.24% (30/34), sensitivity 90% (9/10), specificity 87.50% (21/24), PPV 75% (9/12) and NPV 95.45% (21/22). The difference was statistically significant (χ^2^ = 18.565, *p* < 0.001), with a kappa value of 0.73.

For Sm resistance, the concordance was 97.06% (33/34), sensitivity 94.74% (18/19), specificity 100% (15/15), PPV 100% (18/18) and NPV 93.75% (15/16). The difference was statistically significant (χ^2^ = 30.197, *p* < 0.001), with a kappa value of 0.94. For levofloxacin (Ofx) and moxifloxacin (Mfx) resistance, the concordance was 100% (34/34), with sensitivity, specificity, PPV and NPV all at 100%. The difference in detecting Ofx resistance was statistically significant (χ^2^ = 34.000, *p* < 0.001), with a kappa value of 1.00.

For Am, Km and Cm resistance, the concordance was 97.06% (33/34), sensitivity 100% (2/2), specificity 96.88% (31/32), PPV 66.67% (2/3) and NPV 100% (31/31). The difference was statistically significant (χ^2^ = 21.958, *p* < 0.001), with a kappa value of 0.78. The MALDI-TOF MS results showed strong agreement with the MGIT results (*p* < 0.001), indicating that the new assay’s performance was significantly better than chance and closely aligned with the gold standard.

### 3.4 Drug-resistant gene mutations

Among the 100 retreatment patients, the nucleic acid-based MALDI-TOF MS assay detected drug resistance-associated gene mutations. This detection rate of 45% (45/100) reflects the proportion of patients in whom drug resistance mutations were identified, which is characteristic of the retreatment TB population studied rather than an indicator of test performance per se. Among the 45 drug-resistant cases, the multidrug resistance (MDR) rate was 82.22% (37/45) (Table 4).

**Table 4:** Statistics of different drug resistance types (*N* = 45).

Type of drug resistance	Cases of drug resistance strains (*n*)	Proportio*n* (%)
Total	45	
Single-drug resistance	8	17.78%
RIF	4	8.89%
INH	2	4.44%
Sm	1	2.22%
PZA	1	2.22%
Dual-drug resistance	9	20.00%
RIF+INH	7	15.56%
RIF+Sm	1	2.22%
RIF+EMB	1	2.22%
Triple-drug resistance	12	26.67%
RIF+INH+Sm	7	15.56%
RIF+INH+EMB	2	4.44%
RIF+EMB+Sm	1	2.22%
RIF+OFX+MFX	2	4.44%
Quadruple-drug resistance	6	13.33%
RIF+INH+Sm+PZA	3	6.67%
RIF+INH+EMB+PZA	3	6.67%
Multi-drug resistance (>4 drugs)	10	22.22%
RIF+Sm+EMB+OFX+MFX	1	2.22%
RIF+INH+Sm+EMB+PZA	1	2.22%
RIF+INH+Sm+EMB+OFX+MFX	2	4.44%
RIF+INH+Sm+PZA+OFX+MFX	1	2.22%
RIF+INH+Sm+EMB+PZA+OFX+MFX	2	4.44%
RIF+INH+EMB+PZA+Am+Km+Cm	1	2.22%
RIF+INH+Sm+EMB+PZA+Am+Km+Cm	1	2.22%
RIF+INH+Sm+EMB+PZA+OFX+MFX+Am+Km+Cm	1	2.22%

RIF, rifampicin; INH, isoniazid; Sm, streptomycin; EMB, ethambutol; PZA, pyrazinamide; OFX, levofloxacin; MFX, moxifloxacin; Am, amikacin; Km, kanamycin; Cm, capreomycin.

Multidrug-resistant (MDR) TB: Defined as resistance to at least both RIF and INH. MDR rate among drug-resistant cases: 82.22% (37/45).

The relevant resistance genes are shown in Table 5, which reveals that the most frequent were RIF-associated rpoB gene mutations, accounting for 91.11% (41/45). Isoniazid-related katG, inhA and ahpC gene mutations accounted for 80.00% (36/45). Streptomycin-related rpsL gene mutations accounted for 48.89% (22/45), and EMB-related embA and embB gene mutations accounted for 37.78% (17/45). Fluoroquinolone-related gyrA gene mutations accounted for 20.00% (9/45), pyrazinamide-related pncA gene mutations for 31.11% (14/45) and amikacin-, kanamycin- and colistin-related rrs gene mutations for 8.89% (4/45). The resistance gene and mutation loci results are presented in Table 6.

**Table 5:** Resistance-related genes for different types of drug resistance (*N* = 45).

Type of drug resistance	Related genes	Cases (%)
Total		45 (100%)
Rifampicin	rpoB	41 (91.11%)
Isoniazid	katG	31 (68.89%)
	inhA	4 (8.89%)
	ahpC	1 (2.22%)
Streptomycin	rpsL	22 (48.89%)
Fluoroquinolones	gyrA	9 (20.00%)
Ethambutol	embB	16 (35.56%)
	embA	1 (2.22%)
Pyrazinamide	pncA	14 (31.11%)
Amikacin/Kanamycin/Capreomycin	rrs	4 (8.89%)

Multiple mutations can occur in a single case; therefore, percentages may sum to more than 100%.

**Table 6:** Resistance genes and mutation sites of different drug-resistant strains (*N* = 45).

Resistance genes and mutation sites	Number of detections
Single gene mutations	
rpoB526	1
rpoB531	2
rpoB533	1
katG315	2
rpsL43	1
pncA156	1
Dual gene mutations	
rpoB531+katG315	1
rpoB526+katG315	1
rpoB533+katG393	1
rpoB457+katG397	1
rpoB531+katG343	1
rpoB513+katG315	1
rpoB531+ahpC	1
rpoB512+rpsL43	1
rpoB526+embB306	1
rpoB450+gyrA94	1
rpoB531+gyrA90	1
Triple gene mutations	
rpoB511+katG315+embB306	1
rpoB513+katG315+rpsL43	1
rpoB514+katG315+rpsL43	1
rpoB526+katG315+rpsL43	2
rpoB531+katG315+rpsL43	2
rpoB531+katG397+embB311	1
rpoB531+embB306+rpsL43	1
Quadruple gene mutations	
rpoB507+katG234+embB318+pncA159	1
rpoB512+katG234+embB297+pncA159	1
rpoB526+katG315+rpsL526+pncA43	1
rpoB531+katG315+rpsL43+pncA97	1
rpoB531+katG315+rpsL46+pncA132	1
rpoB531+katG315+rpsL43+rrs	1
rpoB531+katG315+embB306+pncA131	1
rpoB513+embB406+gyrA90+rpsL88	1
Multiple gene mutations (≥5 genes)	
rpoB531+katG315+embB306+rpsL43+pncA47	1
rpoB531+katG315+embB306+pncA47+gyrA94	1
rpoB531+katG315+pncA59+pncA59+gyrA90	1
rpoB531+katG315+embB88+rpsL88+pncA12+rrs	1
rpoB531+katG315+inhA+embB306+pncA171+rrs	1
rpoB406+katG315+embB531+embA+rpsL43+gyrA94	1
rpoB531+katG315+inhA+embB306+rpsL43+pncA139+gyrA94	2
rpoB531+katG315+rpsL43+embB406+gyrA94+pncA46+rrs	1

Numbers after gene names indicate codon positions. The most common mutation sites were rpoB531, rpoB526, and katG315, consistent with known hot spots for rifampicin and isoniazid resistance.

## 4 Discussion

In this study, we demonstrated that nucleic acid-based MALDI-TOF MS (MassARRAY®) is a promising tool for the rapid identification of MTB and the detection of drug resistance in patients with retreatment pulmonary TB. The findings underline the potential clinical value of this nucleic acid-based technology in improving diagnostic efficiency and supporting more personalized treatment regimens.

The relatively lower specificity (63.64%) observed in our study may be attributed to several factors. First, the low bacterial load in BALF samples may lead to the nucleic acid-based mass-spectrometry detection threshold being set with higher sensitivity, potentially increasing false-positive results. Second, mixed infections with NTM or other bacteria could contribute to false-positive MTB identification. Third, the MassARRAY® system’s high analytic sensitivity may detect non-viable or fragmented mycobacterial DNA through its nucleic acid amplification process. Among the 12 MALDI-positive/culture-negative cases, 6 were identified as NTM, which may represent true infections that culture failed to detect, highlighting the enhanced detection capability of nucleic acid-based molecular methods compared with conventional culture (Liu et al., 2025). Future improvements could include optimizing quality-control thresholds or combining the nucleic acid-based MALDI-TOF MS assay with other rapid tests, such as Xpert MTB/RIF, to enhance specificity.

The high frequency of mutations in rpoB (RIF resistance) and katG (INH resistance) observed in this study can be attributed to repeated and often inadequate exposure to anti-TB drugs in patients undergoing retreatment. These mutations confer a survival advantage to MTB, allowing the bacteria to persist in the presence of antibiotics. The rpoB mutation often arises in response to long-term RIF use, whereas katG mutations are typically associated with prolonged INH treatment, where insufficient dosing or treatment interruptions lead to resistance (Seifert et al., 2015; Miotto et al., 2018). These findings highlight the critical need for targeted treatment strategies, especially in retreatment cases where drug resistance is more prevalent.

Our results align with those of Li et al. (2022), who used nucleic acid-based MALDI-TOF MS to detect drug-resistant MTB in new TB cases, reporting similar sensitivity and specificity. However, our study differs in that it focuses on patients with retreatment TB – a group with a higher prevalence of drug resistance. This distinction is important, as retreatment cases are more likely to harbor MDR or XDR strains and thus may benefit more from the rapid diagnostic capabilities of nucleic acid-based MALDI-TOF MS. Li et al. (2022) observed that nucleic acid-based MALDI-TOF MS performed well in terms of species identification but had lower specificity in identifying drug-resistant strains, particularly in areas with mixed mycobacterial populations. In contrast, our study showed that nucleic acid-based MALDI-TOF MS was effective in both identifying MTB and detecting drug resistance in retreatment cases, though specificity for certain resistance mutations (e.g., INH) still requires optimization (Luo et al., 2023; Dekkers et al., 2019).

Furthermore, our findings support those of Neuschlova et al. (2017), who compared MALDI-TOF MS with conventional methods, such as Xpert MTB/RIF and MGIT 960 culture. Although Xpert MTB/RIF provided rapid results, its sensitivity for drug resistance detection was lower than that of culture-based methods. Our study demonstrates that nucleic acid-based MALDI-TOF MS could complement these methods by offering faster results without compromising diagnostic accuracy, particularly in high-burden regions where time is critical for managing drug-resistant TB (Izudi et al., 2023; Rocha et al., 2021; Xia et al., 2020; Kontsevaya et al., 2024).

## 5 Limitations

This study has several limitations. First, the sample size of 100 patients from a single center limits the generalisability of our findings. A larger, multicentre study would provide more robust evidence for the clinical utility of nucleic acid-based MALDI-TOF MS in patients with retreatment TB. Second, the retrospective design may introduce selection bias, and a prospective study would better control for confounding factors. Third, the lower specificity of nucleic acid-based MALDI-TOF MS for certain drug-resistant strains, particularly in cases with low bacterial load or mixed infections with NTM, requires further optimization of the testing protocol. Our team is currently initiating a multicentre prospective study with a target sample of 400 patients to address these limitations.

### 5.1 Integration into clinical practice

The implementation of nucleic acid-based MALDI-TOF MS in clinical practice offers several advantages. According to World Health Organization guidelines on TB diagnostics (World Health Organization, 2021), rapid molecular tests are recommended as initial diagnostic tools for TB detection and RIF resistance testing.

First, nucleic acid-based MALDI-TOF MS can provide rapid diagnosis, complementing conventional methods such as MGIT 960 culture, and delivering results in less than 24 h to support early treatment decisions. Second, the per-sample cost of nucleic acid-based MALDI-TOF MS (approximately USD 60 per sample) is lower than Xpert MTB/RIF (USD 100), which improves accessibility. However, the initial equipment investment for the MassARRAY® system is substantial (approximately USD 300,000-400,000), which may limit accessibility in resource-constrained settings. Therefore, this technology may be most appropriate as a secondary reference or central laboratory tool in tertiary hospitals or regional reference centers rather than a point-of-care test in peripheral health facilities. In high-burden, resource-limited settings, line probe assays or Xpert MTB/RIF may remain more practical options for initial deployment.

Our study showed high sensitivity (97.01%) for MTB detection and excellent agreement with MGIT 960 for drug resistance detection (*κ* = 0.83). The lower specificity (63.64%) primarily reflects detection of cases that culture fails to identify, rather than false negatives. Nevertheless, nucleic acid-based MALDI-TOF MS should be used as a complementary tool rather than a complete replacement for culture-based methods. Culture remains essential for confirming diagnoses, particularly in cases with discordant results, and for phenotypic drug susceptibility testing of drugs not covered by molecular assays.

Integration with other diagnostic tools, such as PCR-based assays or whole-genome sequencing, could further enhance diagnostic accuracy. Unlike stand-alone PCR assays, nucleic acid-based MALDI-TOF MS combines primer extension with accurate m/z discrimination, enabling multiplex detection (greater than 40 loci) in a single run.

Compared with other rapid diagnostic tools, nucleic acid-based MALDI-TOF MS offers faster results than culture and potentially more comprehensive multidrug detection than Xpert MTB/RIF; however, it requires specialized equipment. Line probe assays are better suited for resource-limited settings, whereas next-generation sequencing provides a more complete resistance profile at a higher cost. The MassARRAY® platform occupies an intermediate position between these extremes, offering a reasonable balance of cost and comprehensive drug resistance detection for reference laboratory settings.

### 5.2 Future directions

Future research should focus on the following: (1) conducting multicentre studies to validate our findings across diverse populations and settings, (2) prospective studies to reduce bias and provide more reliable evidence, (3) protocol optimization to improve nucleic acid-based MALDI-TOF MS specificity and sensitivity, particularly for low bacterial load samples; and (4) clinical trials to assess the impact of rapid drug resistance detection on treatment outcomes and public health. The ability of MassARRAY® to distinguish MTB from NTM through species-specific polymorphisms in conserved genes represents a significant advance in mycobacterial diagnostics.

## 6 Conclusion

This study demonstrated that nucleic acid-based MALDI-TOF MS (MassARRAY®) shows promising performance in rapidly identifying MTB, with a high degree of concordance with MGIT 960 culture. Given its strong potential in drug resistance detection, the nucleic acid-based MALDI-TOF MS assay may be considered as a complementary tool alongside conventional culture methods for *Mycobacterium* identification and detection of unknown drug resistance in clinical practice to support individualized treatment plans for patients with retreatment TB. However, considering equipment costs and infrastructure requirements, it is most appropriate for implementation in reference laboratories and tertiary care centers rather than as a complete replacement for established methods. Its application for NTM detection requires further validation with larger sample sizes.

## Data Availability

The original contributions presented in the study are included in the article/supplementary material, further inquiries can be directed to the corresponding author.
